# Deciphering microbial gene function using natural language processing

**DOI:** 10.1038/s41467-022-33397-4

**Published:** 2022-09-29

**Authors:** Danielle Miller, Adi Stern, David Burstein

**Affiliations:** The Shmunis School of Biomedicine and Cancer Research, George S. Wise Faculty of Life Sciences, Tel-Aviv University, Tel-Aviv, 6997801 Israel

**Keywords:** Protein function predictions, Genome informatics, Metagenomics, Machine learning

## Abstract

Revealing the function of uncharacterized genes is a fundamental challenge in an era of ever-increasing volumes of sequencing data. Here, we present a concept for tackling this challenge using deep learning methodologies adopted from natural language processing (NLP). We repurpose NLP algorithms to model “gene semantics” based on a biological corpus of more than 360 million microbial genes within their genomic context. We use the language models to predict functional categories for 56,617 genes and find that out of 1369 genes associated with recently discovered defense systems, 98% are inferred correctly. We then systematically evaluate the “discovery potential” of different functional categories, pinpointing those with the most genes yet to be characterized. Finally, we demonstrate our method’s ability to discover systems associated with microbial interaction and defense. Our results highlight that combining microbial genomics and language models is a promising avenue for revealing gene functions in microbes.

## Abstract (web summary)

The function of many microbial genes is yet unknown. Here the authors repurposed natural language processing algorithms to explore “gene semantics” and infer function for thousands of genes with defense and secretion systems found to have the most discovery potential.

## Introduction

In the post-genomic era, the volumes of genetic data are rapidly accumulating. In particular, metagenomics, the DNA sequencing of microbial communities directly from their ecosystems, provide access to untapped data encompassing a large diversity of microbes that have never been cultivated in laboratory settings^1,2^. Little is known about the function of a considerable portion of the genes encoded by these microbes. Deciphering the function of uncharacterized genes is a major challenge in microbial genomics today. Such genes potentially hold immense value to biotechnology and medicine as genome manipulation tools, antimicrobials, delivery systems, and more^3–5^.

Experimental and computational studies have demonstrated that the genomic context, i.e., the set of genes residing in proximity to a given gene, bears important information regarding the gene’s function^6–10^. This phenomenon is prominent in prokaryotes, where co-functioning genes are often organized in clusters within the genome. An outstanding example of such genomic loci is CRISPR-Cas systems, which encode a series of genes that confer resistance to foreign genetic elements. While the *cas* gene content varies across different system types, the co-occurrence of subsets of *cas* genes within the CRISPR-Cas loci is a strong genomic signature of the system^10–13^.

Using context to infer meaning is a key concept in the field of natural language processing (NLP). Many models applied to natural languages, such as English, use the context of words in a sentence to learn its semantics^14,15^. Modern NLP approaches train deep learning algorithms on large corpora of text, such as Wikipedia, news articles, and other field-specific data sources, to provide meaningful numerical representations to words, which allow deciphering their meaning and semantic relationships. These numerical representations, termed “embeddings”, are used in various downstream applications, from topical text classification to chatbots that simulate conversation. Recently, NLP-based approaches have been applied to model “protein languages”, i.e., to predict properties of amino acids based on their context within a corpus of sequences belonging to a specific protein family. Such applications have been used to model various protein characteristics^16–19^, discover antimicrobial peptides^20^, and even predict antigens leading to viral escape^21^. A different application aimed at classifying biosynthetic gene clusters using Pfam domains rather than amino acids as input to language models^22^. Other studies applied NLP algorithms to DNA k-mers for taxonomic classification^23^, predicting enhancer-promoter interactions^24^, and chromatin accessibility^25^.

Here, we used NLP on a higher level of representation in an attempt to create a universal model of “gene semantics”. In our model, gene families are “words” that comprise “genomic sentences”. To generate these sentences, we re-annotated and analyzed an extensive dataset of publicly available genomes and metagenome, comprised of more than 2.5 Tera base-pairs of assembled sequence data. We transformed the genetic data into a corpus, adding a layer of abstraction by clustering genes into families. We modeled gene families based on their genomic context to study their “semantics” and to predict the function of tens of thousands of uncharacterized genes. We validated our approach by demonstrating that it recovers correctly recently discovered systems. Finally, we assessed which functional categories have the highest “discovery potential” and highlighted three examples of previously uncharacterized systems revealed by our method.

## Results

### Genomic corpus compilation

**Fig. 1:**
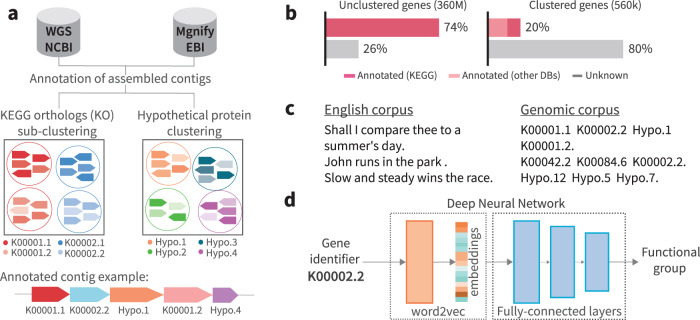
Model workflow. **a** Data processing and annotation. Assembled contigs from public databases were downloaded and underwent gene calling and annotation. Both annotated and unannotated genes were clustered into gene families. **b** Distribution of annotated and hypothetical genes in the corpus. The left bar chart represents all ~360 million genes used in the corpus. The right bar chart represents ~560,000 unique gene families (i.e., the corpus “vocabulary”). **c** A comparison between English and genomic corpora. The “sentences” in the genomic corpus are contigs, which are composed of gene families identifiers as “words”. **d** Embedding generation and function prediction pipeline. Embeddings (numeric vector representations) are generated by the word2vec algorithm and serve as the input to a deep neural network for gene function classification.

Our corpus was compiled from all assembled metagenomes and genomes (excluding green plants, fungi, and animals) publicly available on NCBI’s^26^ and EBI’s^27^ databases. After removing redundancies and short contigs, the dataset contained 11 million contigs, encoding ~360 million genes. Gene family annotation was performed based on KEGG ortholog groups^28^ (Fig. 1a), leading to the annotation of 74% of the genes in our datasets. The remaining genes, lacking a well-defined KEGG annotation, were clustered into gene families based on sequence similarity (Fig. 1b). Each gene family, either annotated or unannotated, with sufficient representation (≥24 genes, see Methods) was considered a “word” in our genomic corpus, resulting in a “genomic vocabulary” of 563,589 words. Notably, even though the annotated genes are the majority of the corpus by counts, after clustering them into families, they comprise only 7.8% of the gene families or unique “words” in the corpus. This means that well-characterized genes with core microbial functions cluster into relatively few, very large families, while most of the genetic diversity (92.2%) in the corpus is not well annotated. In an attempt to annotate genes that were not assigned a KEGG orthology, we searched the NCBI’s non-redundant protein database (NR) and recovered informative annotations (after excluding descriptions such as “hypothetical protein” or “domain of unknown function”) for 14% of the gene families unannotated in KEGG. Overall, ~80% of the gene families had no informative annotation (Fig. 1b and Supplementary Table 3) in either database.

### Gene annotations embedding space

**Fig. 2:**
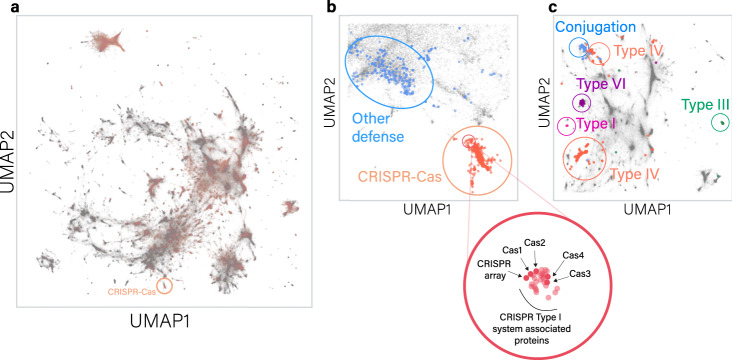
A two-dimensional representation of the space spanned by gene annotation embeddings. **a** Global gene embedding space, including all 563,589 gene families. Each dot in the space represents a gene family, where light red dots represent annotated families and gray dots represent unannotated gene families. The orange circle marks the region that contains most CRISPR-Cas genes (magnified in panel **b** alongside other defense genes). **b** Regions of defense systems clusters marked in circles: *cas* genes are in light red, and light blue dots represent known prokaryotic defense genes. The red circle focuses on the upper CRISPR cluster, enriched with type I CRISPR-Cas system genes. **c** The region encompassing most annotated secretion system clusters color-coded by system type. Source data are provided as a Source Data file.

Intrigued by the high number of unannotated genes, we sought to better understand their function using NLP approaches. Such approaches rely on neural network algorithms that are used to encode words into numeric vectors based on their textual context. These vectors, or “word embeddings”, aim to encapsulate the semantics of words, following the assumption that a word’s context implies its meaning. Here, we transfer the problem of learning a distributed representation of words to learning the same representation for gene function (Fig. 1c). Namely, we analyze genomic regions as sentences composed of genes instead of words. In practice, we trained word2vec^29^, a simple unsupervised neural network model, on the entire gene corpus and used the embeddings learned by the model to create a “gene annotation space” (Fig. 2a). This model captures genetic co-occurrence relationships across our genomic corpus, such that genes with similar contexts will be adjacent in the gene embedding space. Furthermore, similar algebraic relationships between vectors representing gene pairs may imply analogous functional interaction between them (e.g., similar distance and direction between pairs of sensors and regulators, see Supplementary Notes and Supplementary Table 1).

We first examined the embedding of the functionally annotated genes. Reassuringly, genes with similar functions tend to cluster together in the gene annotation space. For example, almost all the *cas* (CRISPR-associated) genes were clustered together in the embedding space and were located in the vicinity of other prokaryotic defense systems. Although located nearby in the embedding space, the CRISPR-Cas cluster was clearly separable from other prokaryotic defense clusters (Fig. 2b), in line with the current understanding of defense islands^10^. We also found clusters representing functional groups such as secretion systems, which were clustered distinctly by system type, and even preserved evolutionary relationships to a certain extent: e.g., type IV secretion systems had a cluster intersecting conjugation systems with which it shares ancestry^30^ (Fig. 2c). Overall, we find that genes of known function tend to cluster together in the high dimensional space we created.

Although genes with similar annotations were most often clustered in close proximity in the gene space, there were some interesting deviations. We found genes with identical annotations that were distant from each other in the embedding space, implying that these gene families might operate in different genomic contexts. For example, unlike most *cas* genes, not all *cas4* “words” were located within the CRISPR-Cas region. They were found in four main distinct clusters in the gene embedding space (Supplementary Fig. 6). While the largest cluster resided within the CRISPR-Cas region, two additional clusters were close to restriction-modification genes, and another small cluster fell next to multiple unknown genes in our embedding space. This can be explained by recent findings suggesting that genes from the *cas4* family have both CRISPR-associated and non-CRISPR-associated functions^31^. This exemplifies the ability of our approach to capture different functions carried out by genes that have the same annotation.

### Embedding-based functional classification

**Fig. 3:**
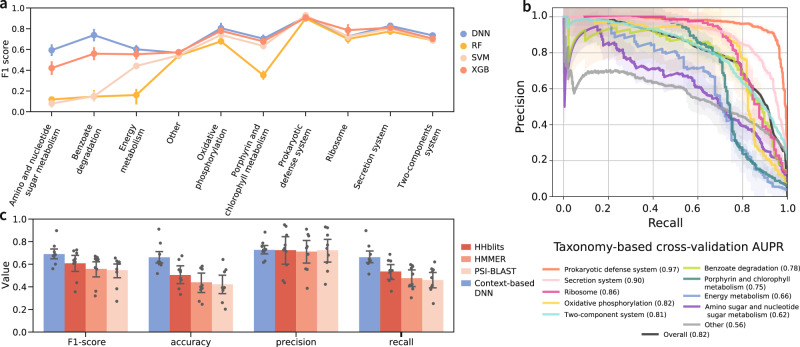
Embedding-based function prediction performance assessment and benchmarking. **a** Classifier comparison. F1 scores were obtained for each category using the leave-one-taxonomic-group-out cross-validation. Each dot represents the average with error bars of ±1 SD obtained from the *n* = 5 folds. DNN deep neural network, RF random forest, SVM support vector machine, XGB XGBoost. **b** Precision–recall curves per functional category calculated using leave-one-taxonomic-group-out cross-validation (see also Supplementary Fig. 2). The “Overall” group refers to the micro-average of all categories combined (i.e., aggregating the predictions of all categories to compute the average). The numeric values of the areas under the curves are denoted for each functional category in the figure legend in parenthesis. Each category line presents the micro-average of the cross-validation folds with ±1 SD. **c** Comparison of our approach against remote-homology search approaches, based on leave-one-KO-out cross-validation. Evaluation metrics were obtained for each of the nine functional categories that are indicated by gray dots. Bar height is the average score with error bars of ±1 SD. Source data are provided as a Source Data file.

Next, we wished to use the embeddings of annotated genes to train a model for gene function prediction. We used KEGG’s functional hierarchy to label each annotated gene with one of ten functional categories (specified in Fig. 3). With the gene embeddings as input, we trained four classifiers: support vector machine^32^ (SVM), random forest^33^, XGBoost^34^, and deep neural network^35^ (DNN) to assign genes to one of the functional categories. We assessed the performance of the classifiers using a taxonomy-based cross-validation. The genomic data set was divided into five major taxonomic groups, and we iteratively tested the ability to accurately predict the functional category of the genes in one major taxonomic group based on an embedding and a classifier trained on the remaining four groups (see Methods). This procedure assesses the model’s performance on unseen, evolutionary-distant genomes, and we refer to it as leave-one-taxonomic-group-out cross-validation. The best-performing algorithm overall in terms of classification performance and speed was a DNN model (Fig. 3a and Supplementary Fig. 4). The classifier’s performance per functional category was high for most categories with area under the precision–recall curve (AUPR) values of 0.56–0.97 (Fig. 3b and Supplementary Fig. 2). These results demonstrate the effectiveness of contextual-based functional inference even across large evolutionary distances, as the classification was based solely on genomic context, without considering gene sequence or any other external information.

The prediction results demonstrate that functional information captured by the embeddings is not uniform throughout all categories (Fig. 3 and Supplementary Table 4). For example, the embeddings are highly informative for the classification of secretion systems, prokaryotic defense systems, and oxidative phosphorylation genes. These functions typically have strong contextual constraints, are composed of multiple genes, and their genes seldom belong to other functional pathways. In contrast, gene embedding was less informative for functional categories such as amino sugar metabolism. Generally, systems lacking a contextual signature and genes associated with multiple different pathways hinder the classifier’s objective.

Given the high prediction performances of our approach, we wished to compare them with sequence-based remote-homology search algorithms. To that end, we benchmarked our approach on the KEGG orthology dataset against three well-established search approaches: PSI-BLAST^36^, HMMer^37^, and HHblits^38^. For the comparison, we performed cross-validation on the level of KEGG’s orthologous families (KOs) for the genes belonging to the ten major functional categories: 2915 separate models were trained, each leaving a single orthologous family out. The predictions were compared to the sequence-based search results after omitting “self hits” (hits to the query’s KO). Overall across the different functional categories, our NLP approach was most often better or comparable to the most sensitive homology-based tool, HHblits, despite relying solely on the gene coordinates in the embedding space (Fig. 3c and Supplementary Dataset 4). The embedding-based classification was, on average, 1.4 times more sensitive than the best-performing homology-based approach, as 27–44% of the tested genes did not have a significant hit in the sequence search algorithms. We note that the precision of HHblits for some functional categories was higher by 4–6% as compared to our approach, with slightly fewer false positives, as can be expected when relying on sequence homology.

### Gene embeddings can reveal the function of unannotated genes

**Fig. 4:**
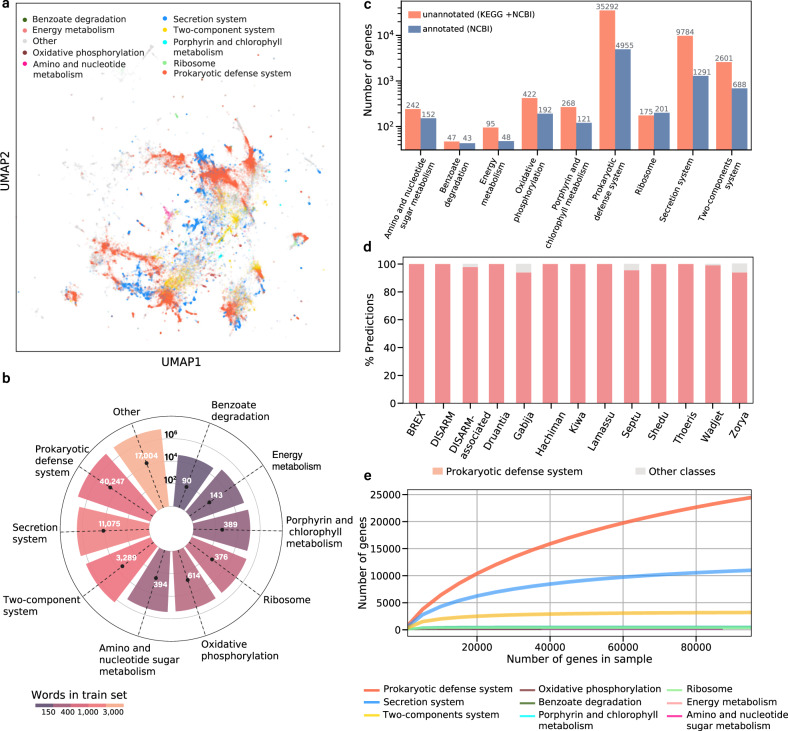
Functional prediction of hypothetical gene families. **a** The complete prediction space. The gene families are color-coded based on the predicted functional categories. **b** Predictions per functional category. Each bar represents the total number of hypothetical genes assigned to a category. The black dot represents the number of hypothetical gene families that received the category prediction. The number of predicted families is explicitly stated next to each dot. Bars are color-coded by the number of words per functional category used to train the model. **c** The number of genes that received reliable predictions in each category, divided into genes with and without informative annotation in NCBI NR. **d** Functional prediction for gene families belonging to recently discovered defense systems. **e** Rarefaction analysis. Each line corresponds to a functional category. The *x*-axis represents the number of sampled genes, and the *y*-axis states the number of gene families with a predicted functional category in the subsample. Source data are provided as a Source Data file.

After training the model on annotated genes, we set out to assign a functional category to the hypothetical genes in our corpus. Overall, our embedding space included 519,398 hypothetical gene families, out of which 444,521 were also unannotated in NCBI’s NR protein database. Using our trained DNN, we predicted the function of 56,617 hypothetical gene families, spanning a total of more than 20 million genes (Fig. 4a, b). We considered only highly reliable predictions, taking into account both the prediction score and the reliability of the classification of each functional category (see Methods).

To further validate our findings, beyond the cross-validation analysis, we investigated the set of 56,617 genes for which our classifier provided high-scoring predictions. These genes were unannotated by KEGG, but for 7691 genes, we were able to recover annotations from BLAST searches against NCBI NR (Fig. 4c). Overall, the gene descriptions agreed well with the model’s prediction. For example, inspecting the two lowest AUPR categories revealed that the predicted “Energy metabolism” genes were indeed associated with this category and its subcategories in the KEGG hierarchy, including numerous genes related to nitrogen and methane metabolism. Similarly, the most abundant description for genes predicted to be in the “Amino sugar and nucleotide sugar metabolism” category were glycosyltransferase and glucosamine-6-phosphate deaminase. The full NR annotations recovered for genes predicted in each functional category are detailed in Supplementary Dataset 7.

Numerous prokaryotic defense systems were recently discovered and experimentally validated. Since the genes associated with these systems are not yet annotated in KEGG and are mostly unannotated in NR, we could utilize this large body of information to further verify our approach. We thus tested our ability to detect genes associated with the BREX, DISARM, Theoris, Durantia, Gabija, Hachiman, Kiwa, Lamssu, Septu, Wadjet, and Zoria anti-phage systems^8,39,40^. Our corpus included 1369 words mapped to genes associated with these systems. Reassuringly, we correctly classified 98.6% of these as prokaryotic defense genes (Fig. 4c, d and Supplementary Table 8). This demonstrates the ability of our approach to detect gene function, even if they share no homology to the annotated genes used to train our classification model. Notably, in addition to the genes of the abovementioned systems, 40,247 uncharacterized gene families were predicted as associated with defense systems, indicating a vast diversity of undiscovered prokaryotic defense systems.

Examining the classifier’s results revealed that the number of gene families varied considerably among the predicted functional categories. To estimate the “discovery potential” of additional genes for each category, we conducted an adjusted rarefaction analysis per category (Fig. 4e). We repeatedly sub-sampled the genes in our dataset and tested how many gene families were predicted in each functional category across different sample sizes. This allowed us to extrapolate how many gene families are still expected to be discovered in each functional category. As anticipated, in well-studied categories associated with core cell functions, such as ribosomal genes and nucleotide metabolism, increasing the number of genes did not bring to light additional gene families (represented by a plateau in the rarefaction plot). However, the functional categories prokaryotic defense system, secretion system, and two-component systems showed a high discovery potential, evident from the continuous rise in gene families predicted in these categories as the total number of genes included in the analysis increased. Indeed, following the conclusion of this research, two large-scale studies discovered 42 prokaryotic defense systems^41,42^, corroborating the high discovery potential of this category. The rarefaction analysis provides a systematic and quantitative assessment of the “discoverability” of yet uncharacterized systems in different functional categories.

### Embedding-based prediction identifies uncharacterized bacterial membrane machineries

Next, we used our predictions not only to detect isolated gene functions but rather clusters of co-occurring hypothetical genes with a similar predicted function. These could reveal previously uncharacterized systems or extended functionality of known systems. We initially focused on genes related to secretion systems, which our rarefaction analysis indicated as a category with high discovery potential. We sought reliable predictions, defined as those with a significant co-occurrence signal across numerous genomes. We identified two such candidates: a postulated secretion-related system and a set of genes highly associated with the type IV pilus system.

**Fig. 5:**
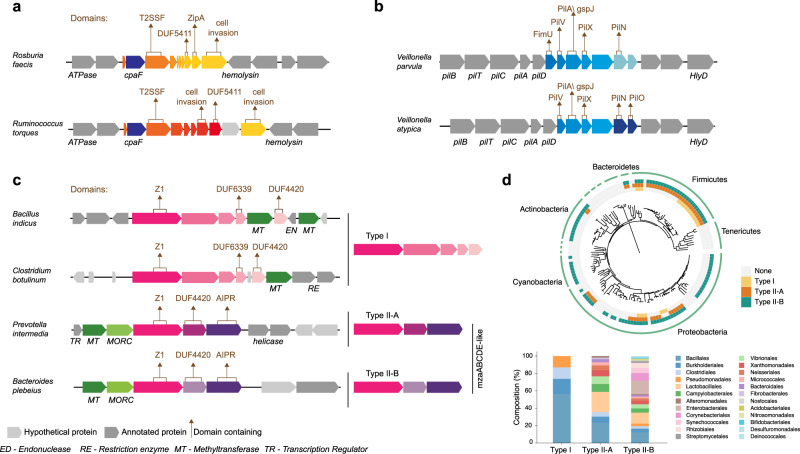
Predicted systems. For all gene operons, known annotations are denoted below the gene illustration, and domains of unannotated genes are marked with arrows above the gene. **a** Predicted secretion-related operons abundant in three Clostridium genera. The genes that were predicted by our approach are marked by the yellow/orange gradient coloring. **b** A distant variant of the type IV pilus system in two representatives of the *Veillonella* genus. The genes predicted by our method are colored in shades of blue. The *pil* genes are annotated type IV pilus genes. **c** Candidate defense system found in multiple bacteria, with representatives from four genomes. **d** The bacterial distribution of the systems presented in **c**. The upper panel includes the bacterial tree of life^70^, color-coded by the presence of each system’s type. The lower panel illustrates the taxonomic distribution of each system on the order level. Source data are provided as a Source Data file.

The first secretion-related system is an operon of eight to nine genes, all labeled by our classifier as secretion system genes. This putative system was prevalent in three genera of the class Clostridia: *Roseburia*, *Ruminococcus*, and *Eubacterium* (detected overall more than 2000 times). The members of these genera are Gram-positive bacteria abundant in the human gut microbiome and affect multiple metabolic pathways in health and disease^43–45^. We observed two main variants, one present in *Roseburia*, and the other in *Ruminococcus* and *Eubacterium*. Both variants share a pilus assembly ATPase (CpaF, a member of the PilB/GspE family), a type IV pilus/type II secretion system membranal protein (PilC/GspF), a protein with the uncharacterized DUF5411 domain, and a protein with cell invasion domain (with remote and partial homology to Internalin-B, Fig. 5a, Supplementary Fig. 7, and Supplementary Table 9). All the other genes did not show a significant resemblance to any known type IV pili/type II secretion components but, in some cases, residing in the vicinity of genes encoding for PilA or adhesion proteins.

The second putative secretion-related operon is encoded in the *Veillonella* genus and is tightly associated with the type IV pili system, which shares close homology with the type II secretion system. This system was found on 86% of *Veillonella* genomes in NCBI’s whole-genome sequence (WGS) database (1280 genomes). *Veillonella* are Gram-negative bacteria of the phylum Firmicutes found in the oral mucosa and intestines of mammals and are known for their ability to interact with other organisms, especially in biofilms, through co-aggregation/co-adhesion. Most *Veillionella* species are associated with the development of common oral diseases^46,47^. Our classifier identified five to six unannotated genes adjacent to IV pili proteins (Fig. 5b, Supplementary Fig. 7, and Supplementary Table 10). This seems to be a distant variant of type IV pili: While five of the genes encode for well-characterized Pil proteins, the genes identified by our classifier are unannotated and share only distant homology to type IV pilus domains, mainly to inner membrane accessory proteins. Notably, we detected homology to all components, excluding the outer membrane PilQ/GspD protein, which was identified hundreds of genes away from that genomic locus. *Veillionella* species are Gram-negative bacteria within the Gram-positive Firmicutes phylum, and this type IV pili system organization and expression may reflect an adaptation to their unique outer membrane^48^.

### Embedding-based classifiers reveal a putative prokaryotic defense system

Our analysis indicated that prokaryotic defense systems have the greatest discovery potential among the functional categories analyzed. We thus used our classification to identify co-occurring uncharacterized gene families that are predicted to have a prokaryotic defense functionality. This led us to discover a putative defense system that contains between three and five uncharacterized genes (Fig. 5c, Supplementary Fig. 8, and Supplementary Table 11). As detailed below, this system encodes various DNA binding and cleaving domains, and it is widespread across the entire bacterial kingdom (Fig. 5d).

We identified two types of this putative system that differ in their gene content, most of which are uncharacterized, although a few contained annotated domains. The core genes of the system, shared by all variants, encode for: (1) a large protein of 925 aa, with a single uncharacterized Z1-domain, which was reported to be associated with restriction-modification systems^49^, (2) a protein with a PD-(D/E)XK motif and a DUF4220 domain, associated with nuclease activity and type II restriction enzymes^50^, and (3) a cytosine methyltransferase. Type I of the system (Fig. 5c) comprised six genes: the three core genes and three additional genes. This type was strongly associated with known DNA repair endonucleases. Type II was divided into two subtypes: II-A and II-B. Both subtypes included the system’s core proteins and an additional AIPR family protein, which is an abortive infection protein^51^. The two subtypes were highly associated with MORC family CW-type zinc finger protein and a putative transcriptional regulator (Fig. 5c). Type II was reminiscent of the recently discovered mzaABCDE system^52^, displaying low sequence similarity (~20% identity) to the mzaBCDE components, and with mzaA (a sigma factor) present only in a few of the systems (the novelty of the systems we identified was also verified with PADLOC^53^ and DefenseFinder^54^). Further investigation is required to determine the system’s function and mechanism. However, the presence of numerous DNA binding and cleaving domains, along with domains shared with the validated mzaABCDE system, suggest that our embedding-based classifier indeed revealed a prokaryotic defense system. The presence of this operonic structure across a very wide taxonomic distribution for both system types further corroborates that this might be a genuine defense system.

## Discussion

We present an approach aimed at universally characterizing functional relationships between microbial genes based on their genomic context. These relationships are captured by the “gene space”, a mathematical representation (embedding) computed according to gene family co-occurrence. In line with the hypothesis underlying this approach, we found that most genes with similar functions tend to cluster together in the gene embedding space. Furthermore, in some interesting cases, variants of the same gene with different functionalities were detected in different regions of the embedding space. We utilized the gene embedding as input for a deep-learning classifier to predict gene function and found that it performs well, with some differential ability to better infer certain functional categories. This is probably due to stronger contextual signals and fewer genes shared among various systems/pathways. Our predictions highlight functional categories with high discoverability potential and reveal unexplored putative bacterial membrane-bound machineries operating in the human microbiome and a microbial defense system, which is widespread in the bacterial kingdom.

NLP approaches have been previously used to characterize genes and proteins, mostly at the level of amino-acid or nucleotide k-mers^16,17,20,22,23^. Here, we targeted a coarser biological level, representing complete genes as the words and exploring their semantics universally. We analyzed an extensive genomic corpus based on all assembled microbial contigs in NCBI’s and EBI’s genomic and metagenomic databases. Our methodology relies on an intuitive adaptation of language models, with genomes as “sentences” and genes as “words”. Encapsulating the raw sequence into gene families reduces noise and increases the abstraction level to one that is relevant for modeling gene function.

In order to process the large volumes of genomic data and ensure meaningful context, we filtered out infrequent genes (families with less than 24 representatives) and short contigs (<10 kbp). Moreover, we used standard gene predictions, which means our analysis does not include small ORFs^55^. This limits our analysis, as rare genes and short peptides are overlooked, and the exclusion of short contigs might hinder the detection of genes on small mobile genetic elements (e.g., transposons and small plasmids). However, the relevant parameters can be tuned for applications aimed to include such elements. For the computation of the gene embedding space, we applied the word2vec algorithm, which is relatively simple, fast, and straightforward. Using more advanced architectures such as transformers and long short-term memory recurrent neural networks could help to further improve the embedding and might better address the sparsity of the genetic data, which includes numerous “unknown words”. These models are not yet optimized for extremely long genomic sequences and require extensive computational resources, yet they hold promise for future gene semantics models.

Our rarefaction analysis indicated that the discovery potential of different functional categories is highly variable. The “saturation” appears mainly in well-characterized categories that belong to the “core genome” of most microorganisms, such as energy metabolism and translation. These functions are expected to contain relatively few uncharacterized genes. However, the apparent “saturation” might also result from methodological limitations discussed above (i.e., lack of contextual signal or genes shared among different systems). Notably, functional categories that are part of the so-called “cloud” or “accessory” genome seem far from saturation. These accessory genes, part of which are associated with defense and secretion, are key to the interactions of microbes among themselves, as well as with their hosts, predators, and environment. As such, many of these genes are in the nexus of evolutionary arms races, leading to their rapid diversification, which can explain the high discovery potential of yet-unknown systems. Notably, accessory genes, and specifically those related to prokaryotic defense and secretion, are often involved in DNA manipulation and virulence^10,42,56,57^ and, as such, may greatly contribute to the development of biotechnological tools and clinical applications.

The implementations presented here barely scratch the surface of the potential that lies in using NLP approaches to “read” genomes. We used the embedding to classify genes for a set of predefined, general, functional categories. However, focusing on domain-specific annotation can be used for in-depth investigations of specific systems or functions of interest. This can be achieved either by using the embeddings provided here, with some fine-tuning, as input to classifiers trained on particular genes of interest, or alternatively by creating a de novo embedding based on relevant corpora, such as viral genomes, specific microbiomes, or on vocabulary that includes additional sets of words (e.g., short peptides or non-coding RNAs). Functional classifiers could also benefit from combining gene embeddings with sequence- and structure-based features, creating models considering both content and context of genes of interest.

The methodology we present has the capacity to significantly enrich our knowledge of microbial gene function. It is uniquely suited to infer the function of genes with no sequence similarity to characterized proteins. It can detect analogous genes, carrying out similar functions without sharing sequence similarities, and highlight dissimilar functions of homologous genes, revealing previously unknown gene specialization. The NLP models describing the “genomic language” can be further enriched: future models could incorporate, in addition to co-occurrence, also information regarding the co-directionality and distance between genes, as well as additional elements, such as promoters, terminators, and regulatory elements as “punctuation” of genomic sentences. We anticipate that the results presented here, coupled with improved and richer models of gene semantics, will lead to a better understanding of gene function and evolution in the vast microbial universe.

## Methods

### Dataset compilation and initial gene annotation

We downloaded all genomes, excluding Metazoa, Fungi, and Viridiplantae, as well as all the metagenomic assemblies that were publicly available in NCBI WGS^26^ and EBI Mgnify^27^ on March 14, 2020. Overall, the dataset included 596,338 genomes and 22,923 metagenomes. These assemblies represent a large phylogenetic diversity and various ecosystems, but they are highly biased toward human-associated microbes (see Supplementary Fig. 1 for taxonomic distribution of genomes and Supplementary Table 2 for a breakdown of the samples used). To remove contigs with little contextual data on the gene level, we filtered out contigs smaller than 10 kbp. As can be expected, this affected the metagenomic assemblies considerably more than the genomic ones: only 26.4% of metagenomic sequence data passed this threshold (530 Gbp were retained out of an initial dataset of 2.01 Tbp), compared to 95.1% of the genomic contigs (2.14 Tbp of an initial 2.25 Tbp dataset). Genes were predicted with prodigal version 3.0.0^58^, and initial gene annotation was performed using the Prokka pipline^59^ (version 1.14.6). To remove duplicated genomes and reduce biases in microbial composition, we used only genomes that mapped to Uniprot’s non-redundant proteomes^60^. This set is comprised of genomes that were clustered on the species level and underwent manual and automatic curation to eliminate redundancies^61^. For the metagenomes, we applied BBMAP dedupe utility^62^ (version 38.69) to remove highly similar contigs. Following these filters, the dataset included 394,374,454 genes encoded on 11,119,550 contigs.

Functional annotation was performed based on KO^63^. Specifically, a total of 17,107,806 proteins from 24,307 KOs were downloaded from the KEGG database on May 14, 2021. The proteins associated with each KO were further subclustered using mmseqs2 cluster^64^ with parameters -s 7.5 -c 0.5. Each subcluster with more than five KEGG proteins was aligned with MAFFT^65^, and the alignments were used to construct a profile HMM using the HMMer suite^37^ version 3.3.2. In total, the KO HMM database included 63,234 HMMs representing 23,199 KOs.

### Corpus generation

To generate the gene corpus, proteins encoded by the non-redundant set of contigs were scanned using the KO HMM database described above. Proteins significantly matching a KO HMM (using hmmsearch with an *E*-value threshold of 10^−6^) were assigned an identifier according to the best scoring KO subcluster in the form of KXXXXX.YY, where KXXXXX is KEGG’s KO and YY is the subcluster index. Proteins unmapped to KOs were considered “hypothetical proteins” and iteratively clustered based on amino-acid sequence similarity as follows. First, highly similar hypothetical proteins were clustered using CD-HIT^66^ with parameters -s 0.80 -c 0.80. Second, CD-HIT representatives were clustered into gene families using mmseqs2^64^ with the same parameters used to subcluster the KOs (-s 7.5 -c 0.5). Each cluster of “hypothetical proteins” was assigned an identifier of hypo.clst.ZZZZ, where ZZZZ is a unique cluster index. Rare gene families (tokens) were filtered out with a threshold of at least 24 appearances per family after testing different threshold values (see Supplementary Table 3). Ubiquitous tokens that appeared in a frequency greater than 10^−^^3^ were also filtered out of the corpus, resulting in a corpus size of 360,039,110 genes represented by 563,589 unique “words”.

We annotated gene families based on KO database because it is extensive and contains detailed metadata and systemic information on the function of each gene family. Yet, not all annotated proteins could be assigned a KO. To assess the number of genes that have a functional annotation but no KO assignment, we searched the representatives of each hypothetical cluster within NCBI’s non-redundant protein database (NR) using DIAMOND version 2.0.11^67^. Proteins were considered unannotated (“hypothetical”) if they had no hit of *E*-value <10^−4^. Proteins were considered to have a reliable annotation if they had significant homology (*E*-value <10^−10^) to a hit with an informative annotation (excluding annotations such as hypothetical proteins, putative functions, and domains of unknown function). Proteins with an *E*-value hit between 10^−4^ and 10^−10^ were considered as proteins with a database hit, but the annotation was not considered reliable for downstream analysis. Following the annotation procedure, each genomic or metagenomic contig was treated as a sentence. The identifier assigned to each ORF, either based on KO subcluster or hypothetical cluster, was used as a word (token) within the sentence.

### Gene space generation

Our goal was to learn a numeric representation, hereby denoted as an “embedding”, to each gene family in our corpus in an unsupervised approach. More formally, let us define $K$ as the set of all KEGG identifiers found in our corpus and $H$ as the set of all hypothetical gene family identifiers. Given a set of tokens $G=({g}_{1},\ldots,{g}_{n})$, such that ${g}_{i}\in K\cup H$, we defined a function $f$ such that $f:{g}_{i}\to {{\mathbb{R}}}^{k},k{\mathbb{\in }}{\mathbb{N}}$. Under the assumption that genomic context can be used to imply gene function, we used the architecture presented in word2vec^29,68^ with the Skip-gram model. This architecture is based on a single-layer neural network and aims to learn the embedding by a simple task: predicting the neighboring words of a given word, i.e., maximizing the log probability $p({g}_{i+j}|{g}_{i})$, where $w$ is the window size selected surrounding the center word ${g}_{i},1\le i\le {|K}\cup {H|}$, $-w\le j\le w{\mathbb{\in }}{\mathbb{N}}$:

1 $$\frac{1}{\left|K\cup H\right|}\mathop{\sum }\limits_{i=1}^{\left|K\cup H\right|}\mathop{\sum}\limits_{-w\le j\le w}{{\log }}\,p\left({g}_{i+j}|\,{g}_{i}\right)$$

In practice, and for speedup, instead of using the softmax function to evaluate $p({g}_{i+j}|{g}_{i})$, we used the negative sampling objective as presented by Mikolov et al.^29^. The final embedding for each ${g}_{i}$ can be obtained as follows:

2 $$f\left({g}_{i}\right)={W}^{T}\cdot {\nu }_{{g}_{i}}$$

Where $\left|K\cup H\right |=n$, ${W}^{n\times k}$ is the weight matrix of the first layer in the network, and ${\nu }_{{g}_{i}}^{n\times 1}$ is a one-hot encoded vector for token ${g}_{i}$. We used a window size $w=5$ and a vector representation of *k* = 300 dimensions per token to describe the “genes embedding space”.

### Embedding-based classification model

We harnessed the pre-trained embeddings of KO tokens to serve as the only features in a classification task (see details on the applied cross-validation below). A simple four-layers DNN was used, with weights ${W}_{i}$, biases ${b}_{i}$, $i\in \left[{{{{\mathrm{0,1,2,3}}}}}\right],$ and hidden layers ${H}_{1},{H}_{2},{H}_{3}$ with sizes 256, 128, and 64 correspondingly. We defined the input embedding matrix ${X \in {\mathbb{R}}}^{{|K|}\times k}$ and the output layer $Y\in {\left[{{{{\mathrm{0,1}}}}}\right]}^{{|K|}\times c}$ for $c$ the number of categories in our classification, which provided label scores for each predicted category (see below the criteria for selecting the categories). The predicted category was defined as follows:

3 $${{{{{\rm{argmax}}}}}}\;{{{{{\rm{softmax}}}}}}({H}_{3}^{T}{W}_{3}+{b}_{3})$$

To avoid overfitting, we added a dropout of 0.2 for all hidden layers. We used ReLU as the activation function for each of the hidden layers and softmax in the output layer. The network was trained with Adam optimizer, categorical cross-entropy loss, and 20 epochs. The number of epochs was chosen according to the loss function convergence, with all other parameters fixed. We tested six different architectures: one or two hidden layers of 256, 128, or 64 neurons, keeping the size of layer *n* – 1 greater than layer *n*.

We also tested SVM^32^, random forest^33^, and Gradient Boosting classifiers^34^. The parameters of each of these models were optimized using a nested five-fold cross-validation grid search on each fold of the training data used for model evaluation. In total, we trained 336 models to explore multiple parameter combinations (Supplementary Table 5), and the best parameter combination was selected based on the F1 score. The best-performing models of each classifier type were: SVM with RBF kernel and regularization of 1 (*C* = 1); random forests with 1000 estimators and maximal depth of 50; and Gradient Boosting classifiers with 800 estimators, maximal depth of 6, and a learning rate of 0.05. Training time comparison of the various models is available in Supplementary Fig. 4 and Supplementary Dataset 4.

The DNN was selected due to its performance and efficiency. To better prioritize its predictions, we assigned a prediction score by weighting the classification score and the accuracy of our model on the relevant function in terms of AUPR. As reliability thresholds, we used *t* = 0.9 for the score weighted according to the AUPR and *t*′ = 0.99 for the non-weighted score.

### Functional categories sampling

KEGG database includes multiple levels of functional annotation of each KO identifier. We used the third-level functional category of the hierarchy of each KO. For KOs associated with multiple functional categories, a single category was chosen based on abundance, relevance to microbial function, and level of detail (table of the KOs and their category assignment is given in Supplementary Dataset 8). The set of all categories $C=\left\{{c}_{1},{c}_{2},\ldots {c}_{n}\right\}$ was reduced into a smaller set for a multiclass prediction, ${C}^{*}=\{{c}_{1}^{*},\ldots,{c}_{m}^{*}\}$, such that ${c}_{i}^{*}\in C$,${c}_{i}^{*}$ had more than 80 appearances in the corpus (the 90th percentile), most of its KOs belonged to one or two functional categories, and its genes have some contextual organization.

The categories that were chosen are amino sugar and nucleotide sugar metabolism (312 genes), benzoate degradation (168 genes), energy metabolism (209 genes), oxidative phosphorylation (409 genes), porphyrin and chlorophyll metabolism (274 genes), prokaryotic defense (871 genes), ribosome (489 genes), secretion systems (1617 genes), and two-component systems (1174 genes); the functional category of each KO is specified in Supplementary Dataset 8. To account for any ${c}_{i}\in {C\backslash }{C}^{*}$, we created a category labeled “Other” by randomly sampling 20 KOs from each category not included in C* that had more than 50 appearances.

### Benchmarking against remote-homology approaches

We compared the function assignment of our method with three widely used sequence-based approaches for remote-homology searches: HHblits, HMMer and PSI-BLAST. For our model, we applied the “leave-one-KO-out cross-validation” procedure described below. For the sequence-based approaches, we searched each word against all words in the dataset that were not assigned the same KO as the query and determined the predicted functional category based on the best hit. HHsuite database was constructed using HHsuite3^38^, and a search was performed using HHblits version 3.3.0 with two iterations and an *E*-value cutoff of 10^–3^. HMMer^37^ version 3.3.2 was used in order to run jackhmmer with two iterations and parameters -N 2 -E 0.001 --incE 0.001. For PSI-BLAST, a BLAST database was constructed using BLAST version 2.7.1, and an iterative search was performed using PSI-BLAST^36^ with two iterations. Results were iteratively filtered for *E*-value <10^–3^ (the evalue-thresh parameter). If no match was found, the predicted category was “no hit”. Hyperparameter selection was based on testing each of the methods with two, three, or four iterations and *E*-value inclusion thresholds of 10^–3^, 10^–4^, or 10^–6^ (see Supplementary Fig. 5, Supplementary Table 6, and Supplementary Dataset 4).

### Cross-validation

To account for evolutionary relatedness while testing the classifiers and to reduce possible biases in the holdout set, we performed two cross-validation procedures on the corpus: (i) naive five-fold cross-validation and (ii) leave-one-taxonomy-group-out cross-validation”. In the naive approach, the entire corpus data were divided into five independent sets leaving out 20% of the contigs. The embedding was computed using word2vec on 80% of the sentences. When testing the classifiers, each gene in the holdout set was searched against the KEGG database to extract the relevant pre-trained embedding, and its functional category was compared to the models’ prediction.

For the taxonomy-aware cross-validation, we used only the genomic data, which included 56,736 genomes (listed in Supplementary Dataset 2). The metagenomic data were excluded to ensure reliable taxonomic mapping. We chose the five most represented taxonomy groups: the Gammaproteobacteria and Alphaproteobacteria classes, and the Actinobacteria, Firmicutes, and Bacteroidetes phyla. Using these five groups, we performed cross-validation (as described above for the naive cross-validation).

To compare remote-homology search algorithms with our approach, we tested their ability to correctly assign functions to each KO using “leave-one-KO-out” cross-validation. We used the same 7043 words applied for performance assessments in the cross-validation described above, such that for all *n* = 2915 unique KOs in the dataset, each fold contained *n* – 1 KOs as a training set, and the words belonging to the remaining KO as a holdout set. We considered in the training and testing dataset all words (subclusters) belonging to the selected KO(s).

For the performance assessment of the cross-validation procedures, we obtained the F1 scores per functional category, the weighted F1 score considering all categories, and the AUPR per category. The per-category AUPR is the micro-average of all folds in a given functional category.

### Rarefaction analysis

To assess the discovery potential according to our predictions, we performed separate rarefaction analyses for each functional category. We defined ${P}_{c}=\{{p}_{1},\ldots,{p}_{k}\}$ as the set of predicted words (hypothetical genes families) for a functional category $c$. Also, we defined ${G}_{c}^{p},{W}_{c}^{p}$ as the word set and word count, correspondingly, of predicted word $p$ in functional category $c$. This quantity refers to the number of genes in the corpus that belong to the family represented by the word $p$. Lastly, we defined ${G}_{c}=\left\{{G}_{c}^{p}{{{{{\rm{|}}}}}}p\in {P}_{c}\right\}$ as the set of all genes predicted for category $c$. We denoted $n\in [{10}^{3},{10}^{6}]$ as the subsample size, such that we uniformly drew $n$ genes from ${G}_{c}$, and obtained the number of unique gene families represented by the subsample (denoted as ${P}_{c}^{n}$). Rarefaction curves were calculated by aligning each subsample of size $n$ with its corresponding total number of sampled gene families ${P}_{c}^{n}$. Confidence intervals were obtained by bootstrapping with 10,000 independent samples.

### Predicted system candidate selection

To seek genes composing yet-unknown systems, we searched for co-occurring gene families with the same predicted functional category that were highly frequent in the corpus and tended to appear together in multiple contigs. Specifically, we selected gene families that occurred more than 1000 times in the corpus and constructed a binary matrix ${{\mathbb{I}}}^{n\times m}$, with $n$ contigs and $m$ selected words, such that ${{\mathbb{I}}}_{{ij}}=1$ if the contig $i$ had the word $j$, and 0 otherwise. This matrix was used to compile a correlation matrix, out of which we extracted highly correlated clusters as systems candidates. Candidate systems were manually selected and explored for domains and remote homology using HHpred^38^ version 57c8707149031cc9f8edceba362c71a3762bd bf8 with default parameters against the databases: PDB mmCIF70 12Oct, Pfam-A v35, NCBI Conserved Domains (CD) v3.18, and TIGRFAMs v15.0. All candidate genes were verified against NCBI’s NR database using BLAST^69^, and the novelty of defense systems gene families was tested against PADLOC^53^ and DefenseFinder^54^.

### Reporting summary

Further information on research design is available in the Nature Research Reporting Summary linked to this article.

## Supplementary information

Supplementary Information

Reporting Summary

## Data Availability

All data in this study were downloaded directly from NCBI WGS and EBI Mgnify on May 14, 2021. The data generated in this study have been deposited in the Zenodo database under accession code 10.5281/zenodo.7047944 (10.5281/zenodo.7047944). These data include representative sequences for each gene family, raw corpus text files, hypothetical family function prediction, gene family mappings to recently reported defense systems, and information on the putative systems identified in this study. Source Data are provided with this paper.

## Source data

Source Data

## Code availability

All the models and scripts developed in this study are available in the repository https://github.com/burstein-lab/genomic-nlp, including links to the relevant data and code used for generating the figures. The paper code release is also available in the Zenodo database under accession code 10.5281/zenodo.7040523 (10.5281/zenodo.7040523). All scripts were written in python ≥3.7. Deep neural networks were conducted in tensorflow version 2.2.0. Machine learning models for performance comparison were trained using sklearn package version 0.24.1 and xgboost version 1.3.3. All figures were generated with seaborn package version 0.10.0 and matplotlib version 3.3.4. Additional python packages used were numpy version 1.20.1 and scipy version 1.2.1.
