# Supplementary material for: Deciphering microbial gene function using natural language processing
Source: Nat Commun. 2022 Sep 29;13:5731. doi: 10.1038/s41467-022-33397-4 (PMC9523054; doi:10.1038/s41467-022-33397-4)
Supplement: Supplementary file 1 — Supplementary Information [file 41467_2022_33397_MOESM1_ESM.pdf]

Deciphering Microbial Gene Function Using Natural Language Processing

Supplementary Material

Danielle Miller[1], Adi Stern[1], and David Burstein[1]

[1]The Shmunis School of Biomedicine and Cancer Research, George S. Wise Faculty of Life Sciences, Tel Aviv University, Tel Aviv, Israel
davidbur@tauex.tau.ac.il, daniellem1@mail.tau.ac.il

Contents

Page No.

Supplementary Notes: Biological analogies 2
Supplementary Datasets 21

Supplementary figures

Supplementary Fig. 1	Genomes per taxonomy distribution.	5
Supplementary Fig. 2	Classifier AUPR using leave-one-taxonomy-group-out cross-validation.	7
Supplementary Fig. 3	Classifier AUPR using naïve cross-validation.	8
Supplementary Fig. 4	Training time comparison for different classifiers	10
Supplementary Fig. 5	Remote homology performance optimization.	11
Supplementary Fig. 6	Cas4 dispersion in a two dimensional representation of the gene embedding space.	14
Supplementary Fig. 7	Co-occurrence of genes in the membrane machineries.	18
Supplementary Fig. 8	Co-occurrence of genes in the novel defense system.	20

Supplementary tables

Supplementary Table 1	Sensor::regulator analogy accuracy by gene family pairs.	4
Supplementary Table 2	Origins of the most abundant metagenomes.	6
Supplementary Table 3	Corpus composition.	9
Supplementary Table 4	Classifier confusion matrix.	9
Supplementary Table 5	Parameter optimization for ML models.	10
Supplementary Table 6	Comparison of running time and performances of our context-based DNN and remote homology methods.	12
Supplementary Table 7	Number of predictions unannotated by KEGG, separated by functional category.	13

Supplementary Table 8 Gene families mapped to recently discovered anti-phage systems. 15
Supplementary Table 9 Predicted membrane machineries in Clostridia 16
Supplementary Table 10 Predicted membrane machineries in *Veillonela* 17
Supplementary Table 11 Predicted prokaryotic defense systems . . . 19

Supplementary Notes

Biological analogies

In standard natural language processing (NLP) models, aimed for languages such as English, word embeddings can be evaluated according to their ability to capture semantic relationships. For example, the analogy Capital::Country, implies that Berlin and Germany have the same relationship as Tokyo and Japan, where '::' notation is used to mark an analogy [1,2]. Here, we define "biological analogies" as similar functional relations between pairs of genes. We were interested in testing whether our embedding vectors can reveal such relationships. This task is not trivial even when applied to English. In our case, additional challenges arise from the fact that a single gene may carry multiple functions, and the interaction within a specific pathway can be complex.

We have compiled a set of 75 pairs of sensors and regulators from 11 families based on the KEGG pathway ko02020: Two-component system https://www.genome.jp/kegg-bin/show_pathway?ko02020 (only pairs of activator-regulator were used). KEGG identifiers were adjusted to fit the form used in the corpus to include all subclusters of each KO, yielding a total of 433 pairs. All pair combinations were generated as a training set S such that:

$$S = \{(< r_i, r_j >, < p_i, p_j >) | r_i, r_j, p_i, p_j \in TC\}$$

Where TC is the set of all two-component system genes selected, represented by their subcluster KOXXXXX.YY. A total of 186,192 analogies were obtained by considering all possible pair combinations, as reference predicted pair. We used the 3CosMul distance metric [3] to evaluate all analogies. Accuracy was obtained for each pair of families separately (for example predicting *OmpR* genes using *CitB* genes).

We found that the accuracy of revealing correct sensor::regulator pairs is strongly dependant on the type of reference and predicted pair, ranging from 0.82 to 0.14 (Table S1).

**Reference family**	**Predicted family**	**Accuracy**
LuxR	*CitB*	0.82
LitTR	*CitB*	0.80
NarL	*CitB*	0.79
OmpR	*CitB*	0.77
CitB	*CitB*	0.75
CitB	*LuxR*	0.51
NarL	*LuxR*	0.38
LitTR	*LuxR*	0.35
OmpR	*LuxR*	0.35
CitB	*OmpR*	0.27
LuxR	*OmpR*	0.24
CitB	*NarL*	0.23
NarL	*OmpR*	0.22
CitB	*LitTR*	0.21
LuxR	*LuxR*	0.21
LitTR	*OmpR*	0.20
OmpR	*OmpR*	0.20
LuxR	*LitTR*	0.17
LuxR	*NarL*	0.16
OmpR	*NarL*	0.16
LitTR	*NarL*	0.16
NarL	*LitTR*	0.14
NarL	*NarL*	0.14
LitTR	*LitTR*	0.14
OmpR	*LitTR*	0.14

Supplementary Table 1: Sensor::regulator analogy accuracy by gene family pairs.

Supplementary information

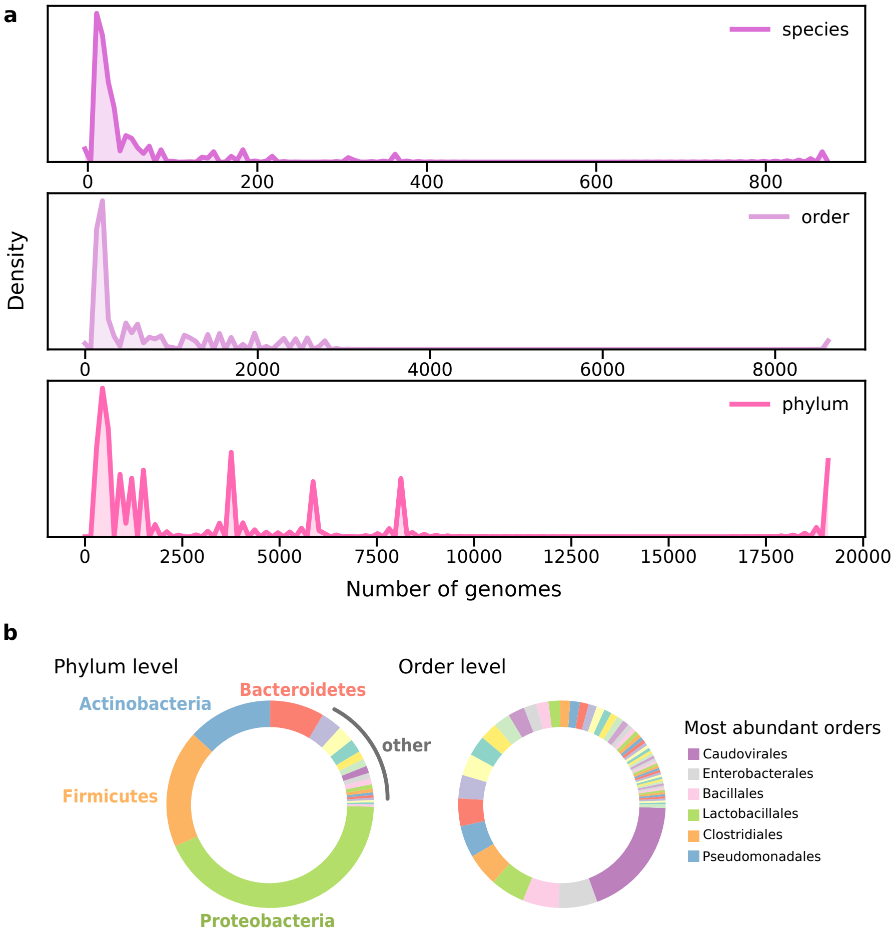

Supplementary Fig. 1: Genomes per taxonomy distribution.

(a) The distribution of genomes with known taxonomy by species (12,554 assembled accessions), order (50,114 genome assemblies) and phylum (48,069 genome assemblies). (b) Genome distribution used in the corpus by phylum and order. Taxa were filtered to maintain the top 0.9 quantile (133, 247 genomes for phylum and order, respectively). All genomes that were filtered out were summed together and are included in the graph as a single group. Full table is available in Dataset 1.

**Metagenomes' origin**	**Number of genes**
Human-associated microbiome	141,203,219
Marine	24,841,394
Unspecified metagenome	20,470,670
Soil	7,944,071
Sludge	7,318,052
Bioreactor	5,445,554
Animal-associated	5,142,375
Rock	4,772,971
Wastewater	3,427,949
Freshwater	2,780,814
Seawater	2,423,172
Sediment	2,315,900
Fermentation	1,148,037
Compost	1,100,998
Hydrothermal vent	874,096
Mine-associated	471,819
Activated carbon	446,632
Microbial mat	415,165
Food	369,311
Other	1,961,285

Supplementary Table 2: Origins of the most abundant metagenomes.

Metagenomes from similar environments were merged into a single category (e.g., human gut and human oral are part of human-associated microbiome). The category 'other' refers to the less frequent metagenome environments, with number of genes as their summation. Full table is available in Dataset 1.

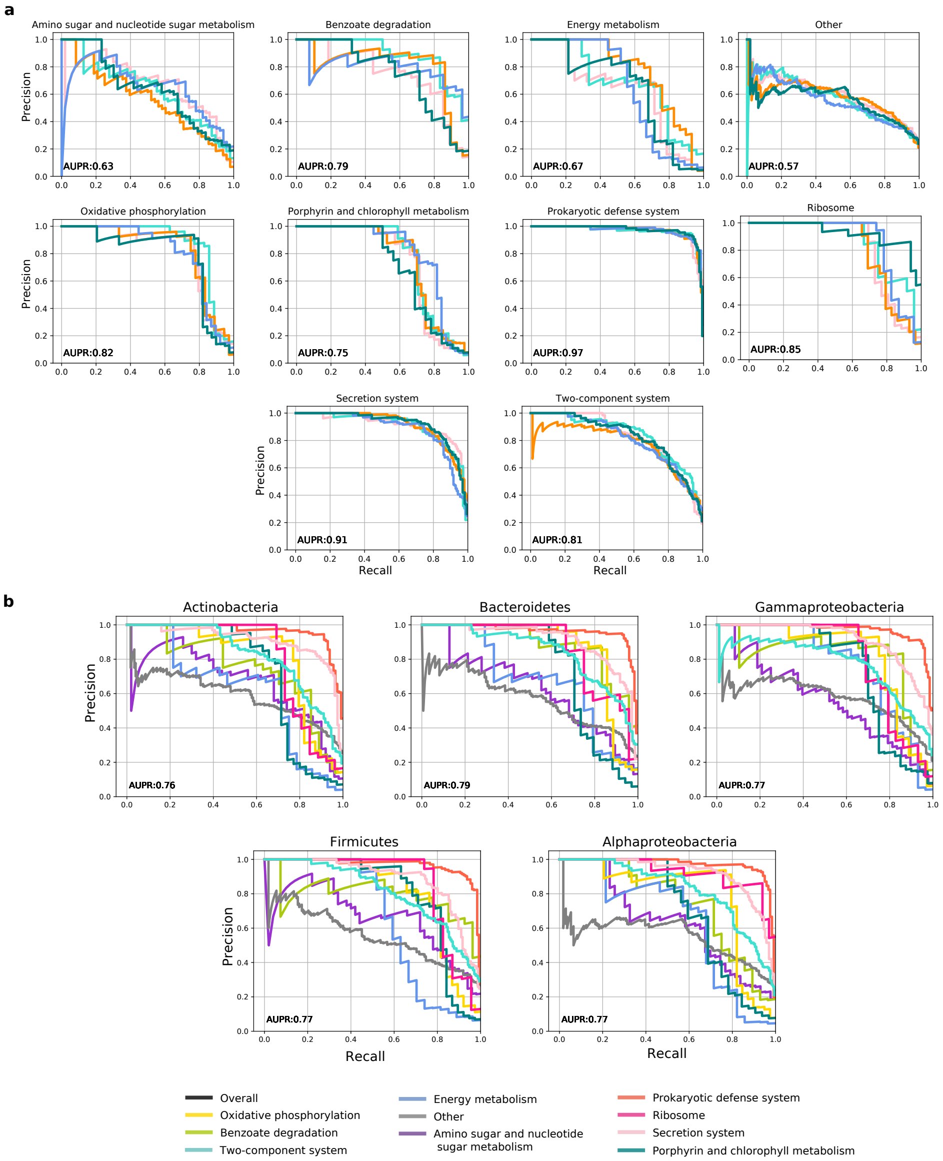

Supplementary Fig. 2: Classifier AUPR using leave-one-taxonomy-group-out cross-validation.

(a) Each functional category is presented in a separate plot. The line colors correspond to each of the to a leave-one-taxonomy-group-out cross-validation fold. (b) Each panel corresponds to a leave-one-taxonomy-group-out cross-validation fold. The taxonomic group that was used as a holdout set is stated in each panel title. The line colors correspond to each of the categories. Embedding model, training data and cross-validation reports are available in Dataset 2.

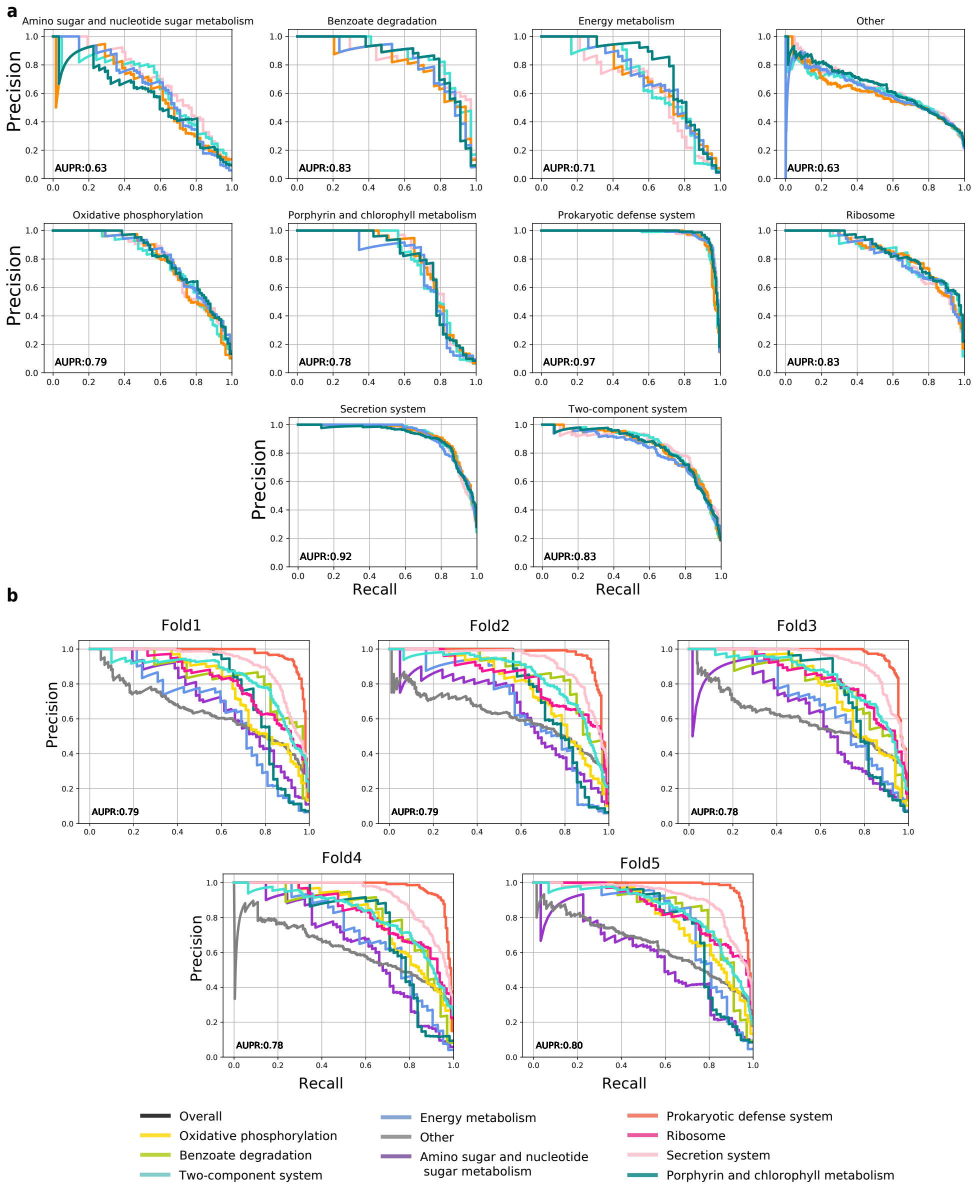

Supplementary Fig. 3: Classifier AUPR using naïve cross-validation.

(a) Each functional category is presented in a separate plot. The line colors correspond to each of the 5-folds used for cross-validation. (b) Each panel corresponds to a fold, such that 80% of the genomes used for embedding generation, whereas 20% of the genomes were used as a holdout set. The line colors correspond to each of the categories. Embedding model, training data and cross-validation reports are available in Dataset 2.

Corpus type	Min gene frequency	Corpus size	Unique tokens	KEGG tokens count	Unique KEGG tokens	Unique hypothetical tokens	Hypothetical tokens count	Tokens mapped to hypothetical proteins in NR	Tokens mapped to annotated proteins in NR	Tokens not found in NR
KO annotation	6	373,208,410	1,758,730	267,329,033	18,769	1,739,961	105,879,377	976,075	204,109	559,777
KO annotation	12	366,989,361	965,230	267,320,580	17,738	947,492	99,668,781	600,582	123,737	223,173
KO annotation	24	360,098,810	536,104	267,303,347	16,706	519,398	92,795,463	361,350	74,877	83,171
KO annotation	50	352,085,758	298,979	267,261,890	15,525	283,454	84,823,868	204,166	45,089	34,199
KO annotation	100	343,199,024	170,883	267,172,296	14,294	156,589	76,026,728	114,443	27,889	14,257
Extended KO annotation	6	373,198,821	1,790,040	267,319,493	50,082	1,739,958	105,879,328	976,075	204,109	559,774
Extended KO annotation	12	366,965,697	994,825	267,296,950	47,335	947,490	99,668,747	600,582	123,737	223,171
**Extended KO annotation**	**24**	**360,039,110**	**563,589**	**267,243,650**	**44,191**	**519,398**	**92,795,460**	**361,350**	**74,877**	**83,171**
Extended KO annotation	50	351,922,327	323,554	267,098,462	40,100	283,454	84,823,865	204,166	45,089	34,199
Extended KO annotation	100	342,801,402	192,207	266,774,677	35,618	156,589	76,026,725	114,443	27,889	14,257

Supplementary Table 3: Corpus composition.

Corpus statistics are presented for two main parameters: corpus type and minimum gene frequency. Corpus type 'KO annotation' refers to corpora with a single token per KEGG Orthologous group (KO), with tokens (i.e., words) labeled with KO identifiers. 'Extended KO annotation' refers to corpora with KO subclustering, with tokens label as KOXXXXX.YY, where KOXXXXX is the KO identifier and YY is the subcluster index. Minimum gene frequency is the minimal number of appearances for a token to be included in the corpus. The corpus used in the study is marked in bold. NR: NCBI's non-redundant protein database. Corpus .txt files are available in Dataset 3.

	Amino sugar and nucleotide sugar metabolism	Benzoate degradation	Energy metabolism	Other	Oxidative phosphorylation	Porphyrin and chlorophyll metabolism	Prokaryotic defense system	Ribosome	Secretion system	Two-component system
Amino sugar and nucleotide sugar metabolism	**36**	0	1	20	1	0	0	1	1	2
Benzoate degradation	0	**26**	0	7	0	0	0	0	0	1
Energy metabolism	0	0	**25**	7	0	4	3	0	1	2
Other	11	10	3	**281**	6	3	7	11	32	28
Oxidative phosphorylation	1	0	2	13	**60**	0	0	3	3	2
Porphyrin and chlorophyll metabolism	0	1	0	13	1	**40**	0	0	0	0
Prokaryotic defense system	0	1	1	9	0	0	**156**	0	5	2
Ribosome	0	0	0	27	2	1	0	**63**	3	2
Secretion system	0	0	0	23	2	1	5	4	**281**	7
Two-component system	2	1	0	50	4	1	0	2	17	**158**

Supplementary Table 4: Classifier confusion matrix.

Confusion matrix of a representative fold. Counts on the diagonal are correct predictions, marked in bold. The table is also available in Dataset 2.

Classifier	Parameters	Number of models tested	Best parameters
SVM	kernel∈ $[linear, RBF]$<br>C∈ $[0.01, 1, 10]$	6	kernel=RBF, C=1
RF	max_depth∈ $[6, 10, 50, 100, none]$<br>max_features∈ $[auto, sqrt]$<br>min_samples_leaf∈ $[1, 2, 4]$<br>min_sample_split∈ $[2, 5, 10]$<br>n_estimators∈ $[100, 500, 1000]$	270	max_depth=50<br>max_features=sqrt<br>min_samples_split=2<br>min_samples_leaf=1<br>n_estimators=1000
XGB	max_depth∈ $[6, 10, 50, 100, none]$<br>learning_rate∈ $[0.001, 0.05, none]$<br>n_estimators∈ $[100, 500, 800, 1000]$	60	max_depth=6<br>learning_rate=0.05<br>n_estimators=800

Supplementary Table 5: Parameter optimization for ML models.

Parameter optimization for machine learning models using grid search. For each classifier, all parameter combinations were tested. Parameters are noted as defined by the sklearn/xgboost packages names. The "none" parameter assignments follow the defaults specified by the package. Precision, Recall, F1-score, and running time for each parameter combination are detailed in Supplementary Dataset 4.

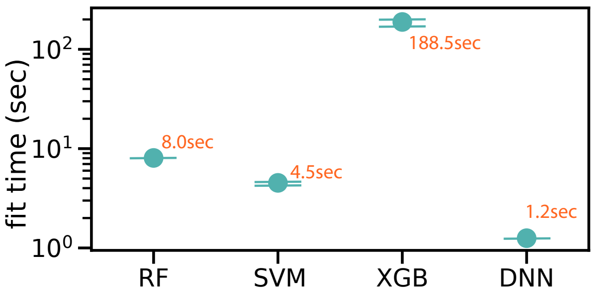

Supplementary Fig. 4: Training time comparison for different classifiers

Training time for each classification method averaged over the 5 folds of the taxonomy-based cross-validation (for 7,043 genes). Error bars are ±1 SD. The average training time is noted near each point. RF: Random Forest, SVM: Support Vector Machine, XGB: XGBoost, DNN: Deep Neural Network. Precision, Recall, F1-score, and running time for each parameter combination are detailed in Supplementary Dataset 4.

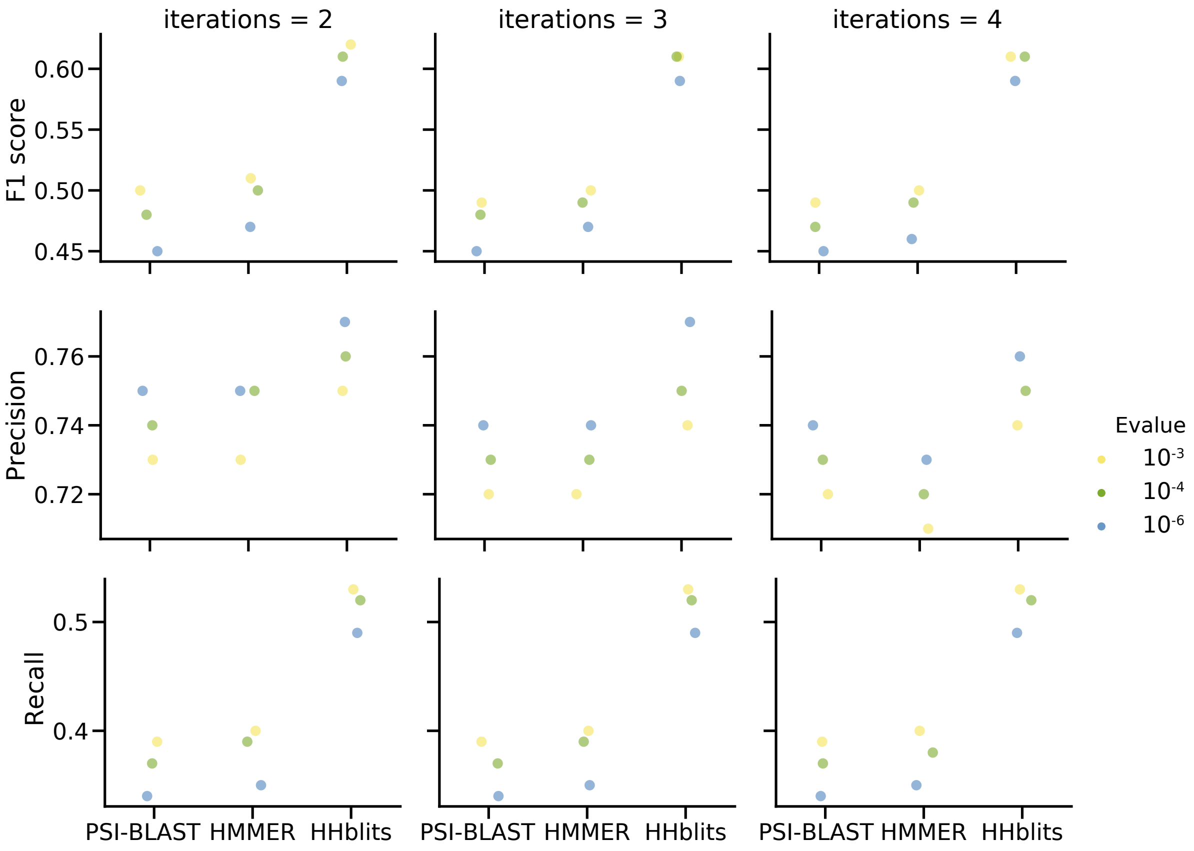

Supplementary Fig. 5: Remote homology performance optimization.

For each remote homology search method, namely PSI-BLAST, jackhmmer from the HMMER suite, and HHblits, we tested E-value inclusion thresholds of 10^{-3}, 10^{-4}, and 10^{-6}, with two, three, and four iterations. The chosen parameters were two iterations and E-value inclusion threshold of 10^{-3}. The tested parameters and their performances are also detailed in Supplementary Table 6 and Supplementary Dataset 4.

Method	Iterations	E-value	Running time (sec)	Precision	Recall	F1-score
Context-based DNN	-	-	0.034	0.7	0.69	0.7
PSI-BLAST	2	0.001	1.48	0.73	0.39	0.5
	2	0.0001	1.47	0.74	0.37	0.48
	2	1.00E-06	1.20	0.75	0.34	0.45
	3	0.001	1.74	0.72	0.39	0.49
	3	0.0001	1.90	0.73	0.37	0.48
	3	1.00E-06	1.29	0.74	0.34	0.45
	4	0.001	1.83	0.72	0.39	0.49
	4	0.0001	1.20	0.73	0.37	0.47
	4	1.00E-06	1.47	0.74	0.34	0.45
HMMER	2	0.001	1.54	0.73	0.4	0.51
	2	0.0001	1.58	0.75	0.39	0.5
	2	1.00E-06	1.88	0.75	0.35	0.47
	3	0.001	1.90	0.72	0.4	0.5
	3	0.0001	2.38	0.73	0.39	0.49
	3	1.00E-06	1.79	0.74	0.35	0.47
	4	0.001	2.22	0.71	0.4	0.5
	4	0.0001	2.24	0.72	0.38	0.49
	4	1.00E-06	2.00	0.73	0.35	0.46
HHblits	2	0.001	15.09	0.75	0.53	0.62
	2	0.0001	14.46	0.76	0.52	0.61
	2	1.00E-06	13.57	0.77	0.49	0.59
	3	0.001	13.68	0.74	0.53	0.61
	3	0.0001	17.27	0.75	0.52	0.61
	3	1.00E-06	19.27	0.77	0.49	0.59
	4	0.001	22.45	0.74	0.53	0.61
	4	0.0001	22.64	0.75	0.52	0.61
	4	1.00E-06	17.17	0.76	0.49	0.59

Supplementary Table 6: Comparison of running time and performances of our context-based DNN and remote homology methods.

For the remote homology methods, running times and performance are noted as a factor of the number of iterations and E-value inclusion thresholds. Running times were calculated on 211 sequences selected from the dataset, preserving the same distribution of sequence lengths. Performances and running times per functional category are available in Supplementary Dataset 4.

**Functional category**	**Mapped to NR**	**Number of predictions**
Amino sugar and nucleotide sugar metabolism	-	242
	+	152
Benzoate degradation	-	47
	+	43
Energy metabolism	-	95
	+	48
Other	-	14881
	+	2123
Oxidative phosphorylation	-	422
	+	192
Porphyrin and chlorophyll metabolism	-	268
	+	121
Prokaryotic defense system	-	35292
	+	4955
Ribosome	-	175
	+	201
Secretion system	-	9784
	+	1291
Two-component system	-	2601
	+	688

Supplementary Table 7: Number of predictions unannotated by KEGG, separated by functional category.

The number of predictions per category that passed the quality thresholds (see Methods). We separate the predictions of genes unannotated in KEGG, into two groups: gene families that were mapped to NCBI's non-redundant database (denoted as 'NR') and genes families that had no annotation, neither in KEGG nor in NR.

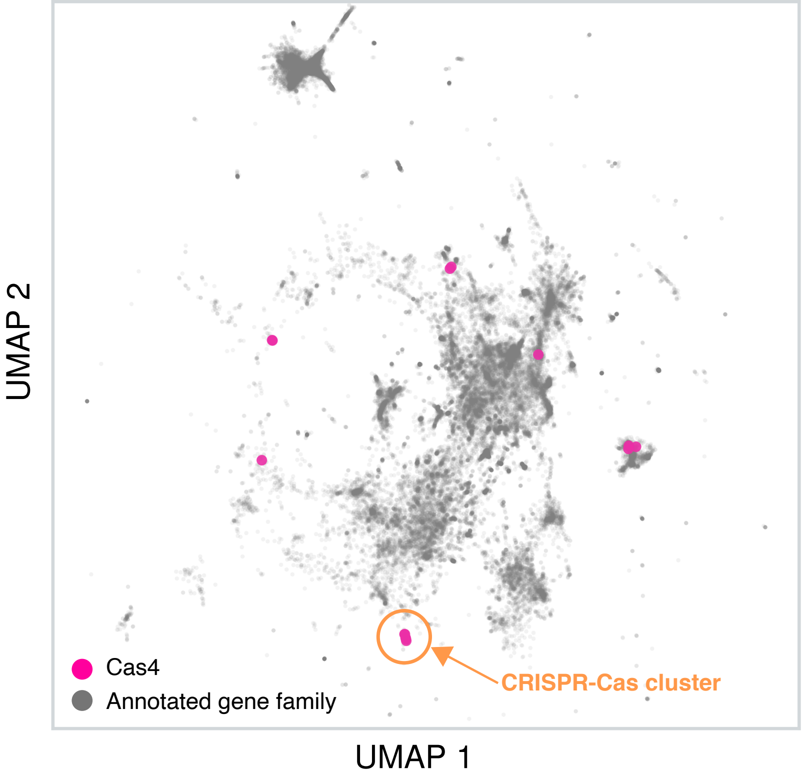

Supplementary Fig. 6: Cas4 dispersion in a two dimensional representation of the gene embedding space.

System	Predicted category	Predictions percentage	Gene family count
BREX	Prokaryotic defense system	100	120
DISARM	Prokaryotic defense system	100	40
DISARM-associated	Prokaryotic defense system	97.83	45
	Ribosome	2.17	1
Druantia	Prokaryotic defense system	100	235
Gabija	Prokaryotic defense system	93.94	62
	Secretion system	6.06	4
Hachiman	Prokaryotic defense system	100	91
Kiwa	Prokaryotic defense system	100	122
Lamassu	Prokaryotic defense system	100	46
Septu	Prokaryotic defense system	95.49	127
	Other	4.51	6
Shedu	Prokaryotic defense system	100	86
Thoeris	Prokaryotic defense system	100	90
Wadjet	Prokaryotic defense system	99.06	210
	Two-component system	0.47	1
	Other	0.47	1
Zorya	Prokaryotic defense system	93.9	77
	Secretion system	3.66	3
	Two-component system	1.22	1
	Other	1.22	1

Supplementary Table 8: Gene families mapped to recently discovered anti-phage systems.

Mapping known, recently discovered, anti-phage systems that are not annotated in KEGG database yielded 1,381 hits. The predicted functional category of the genes belonging to each anti-phage system is presented in the table, including the number of gene families mapped to each category. This table and additional mapping of these systems by gene identifier is available in Dataset 5.

System	Gene family identifier	Protein size	Blast results against NR	Domains (CDD & HHpred)	Homologous gene families	Appearance in other systems
Membrane machineries (*Rosburia*)	hypo.clst.16176103	77	-	-	hypo.clst.10652437	Membrane machineries (*Ruminococcus*, *Eubacterium*)
	hypo.clst.16392502	434	T2SS (only 1 result among hundreds)	PilC		Membrane machineries (*Ruminococcus*, *Eubacterium*)
	hypo.clst.9655398	69	-	-		
	hypo.clst.9511098	35	-	SecY (NS)		
	hypo.clst.580805	48	-	-	hypo.clst.10652437 hypo.clst.9763730	
	hypo.clst.9763730	57	-	-	hypo.clst.580805	
	hypo.clst.3921843	127	-	DUF5411	hypo.clst.719493	
	hypo.clst.7000553	154	Cell division protein ZipA	CpaD		
	hypo.clst.14010549	478	-	Inl-B-internalin, cell invasion		Membrane machineries (*Ruminococcus*, *Eubacterium*)
Membrane machineries (*Ruminococcus*, *Eubacterium*)	hypo.clst.16176103	77	-	-	hypo.clst.10652437	Membrane machineries (*Rosburia*)
	hypo.clst.16392502	434	T2SS (only 1 result among hundreds)	PilC		Membrane machineries (*Rosburia*)
	hypo.clst.10932254	112	-	DUF5411		
	hypo.clst.10652437	35	-	SecY (NS)	hypo.clst.16176103 hypo.clst.6770473 hypo.clst.580805	
	hypo.clst.6770473	151	-	Internalin, cell invasion	hypo.clst.10652437	
	hypo.clst.17543759	24	-	-		
	hypo.clst.719493	68	-	DUF5411	hypo.clst.3921843	
	hypo.clst.14010549	478	-	Inl-B-internalin cell invasion		Membrane machineries (*Rosburia*)

Supplementary Table 9: Predicted membrane machineries in Clostridia

Predicted systems and their gene composition information. Homologous genes share ∼20% sequence identity. NR: NCBI's non-redundant database; NS: non-significant, HHpred E-value > 0.05. Extended table, HHpred domain results, and fasta files with protein sequence is available in Dataset 6.

System	Gene family identifier	Protein size	Blast results against NR	Domains (CDD& HHpred)	Homologous gene families	Appearance in other systems
Membrane machineries (*Veillonela parvula*)	hypo.clst.7011664	141	-	PilV		Membrane machineries (*Veillonela atypica*)
	hypo.clst.17676115	191	-	PilA,GspJ		Membrane machineries (*Veillonela atypica*)
	hypo.clst.10830043	135	-	Pil_X		Membrane machineries (*Veillonela atypica*)
	hypo.clst.6011553	112	-	PilM (NS)		Membrane machineries (*Veillonela atypica*)
	hypo.clst.17539672	112	-	FimU, cell adhesion		
	hypo.clst.4179805	103	Fimbrial assembly protein	PilN		
	hypo.clst.61441	105	-	PilO (NS)	hypo.clst.9647074	
Membrane machineries (*Veillonela atypica*)	hypo.clst.7011664	141	-	PilV		Membrane machineries (*Veillonela parvula*)
	hypo.clst.17676115	191	-	PilA,GspJ		Membrane machineries (*Veillonela parvula*)
	hypo.clst.10830043	135	-	Pil_X		Membrane machineries (*Veillonela parvula*)
	hypo.clst.6011553	112	-	PilM (NS)		Membrane machineries (*Veillonela parvula*)
	hypo.clst.5626151	186	-	PilN		
	hypo.clst.9647074	146	-	PilO	hypo.clst.61441	

Supplementary Table 10: Predicted membrane machineries in *Veillonela*

Predicted systems and their gene composition information in the most abundant *Veillonella* species. Homologous genes share ∼20% sequence identity. NR: NCBI's non-redundant database; NS: non-significant, HHpred E-value > 0.05. Other species of *Veillonella* showed similar patterns, all having the four core genes presented in *Veillonela parvula* and *Veillonella atypica. Veillonella dispar* had additional gene, hypo.clst.61441, and *Veillonella tobetsuensis* had additional two genes: hypo.clst.9647074 and hypo.clst.1980155, the latter was not found in any other species and contains a PilO domain. Extended table, HHpred domain results, and fasta files with protein sequence is available in Dataset 6.

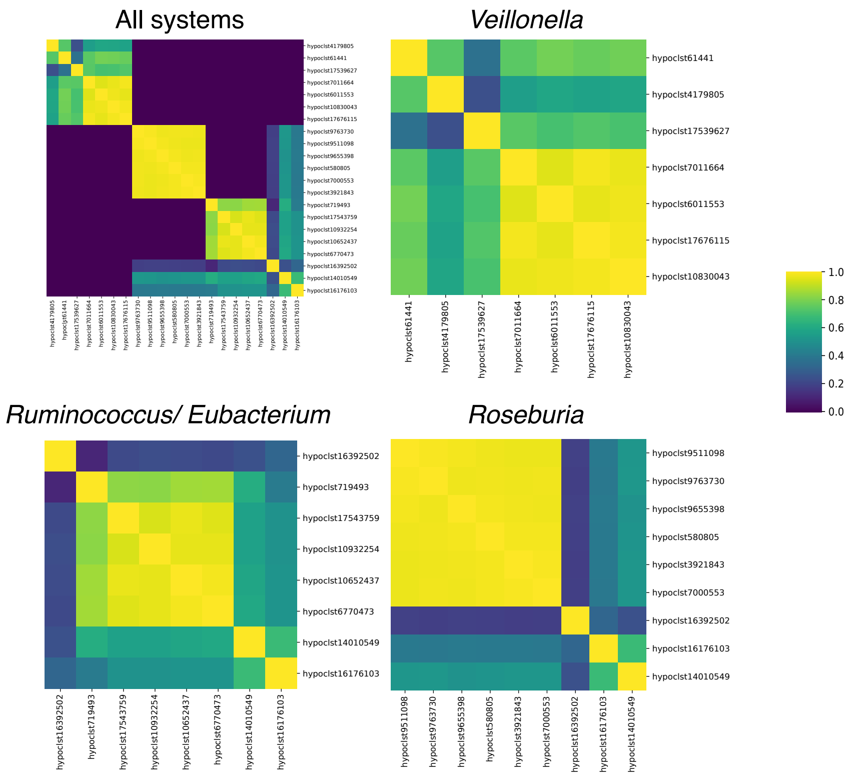

Supplementary Fig. 7: Co-occurrence of genes in the membrane machineries.

Occurrence correlations of the gene belonging to predicted membrane machineries in Clostridia and *Veillonella*.

System	Gene family identifier	Protein size	Blast results against NR	Domains (CDD& HHpred)	Homologous gene families	Appearance in other systems
Type I	hypo.clst.11157878	925	Z1 domain containing	Z1 domain		Type II-A, Type II-B
	hypo.clst.10418776	704	-	MORC family CW-type zinc finger protein 2 (NS)		
	hypo.clst.4335766	298	PD-(D/E)XK motif	DUF4420	hypo.clst.8082052<br>hypo.clst.8193108	
	hypo.clst.5225601	231	-	DUF6339		
	hypo.clst.8286106	310	-	-		
Type II-A	hypo.clst.11157878	925	Z1 domain containing	Z1 domain		Type I, Type II-B
	hypo.clst.15038145	689	AIPR family	AIPR		
	hypo.clst.8082052	319	PD-(D/E)XK motif	DUF4420	hypo.clst.8193108<br>hypo.clst.4335766	
Type II-B	hypo.clst.11157878	925	Z1 domain containing	Z1 domain		Type I, Type II-A
	hypo.clst.15038145	689	AIPR family	AIPR		
	hypo.clst.8193108	295	PD-(D/E)XK motif	DUF4420	hypo.clst.8082052<br>hypo.clst.4335766	

Supplementary Table 11: Predicted prokaryotic defense systems

Predicted systems and their gene composition information. Homologous genes share ∼20% sequence identity. NR: NCBI's non-redundant database; NS: non-significant, HHpred E-value > 0.05. Extended table, HHpred domain results, and fasta files with protein sequence is available in Dataset 6.

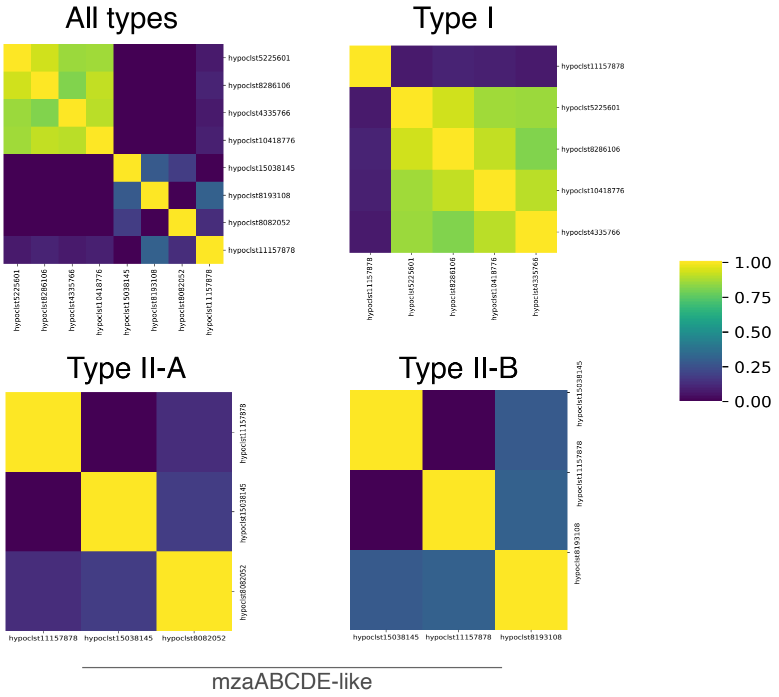

Supplementary Fig. 8: Co-occurrence of genes in the novel defense system.

Occurrence correlation among genes belonging to the predicted defense systems, Type I, Type II-A and Type II-B.

Supplementary dataset description

All data supporting this study's findings will be deposited to the Zenodo database and assigned a permanent DOI; meanwhile they are also available in http://tiny.cc/eb6ouz.

- **Dataset 1** - Taxonomic mapping and metagenomes origins.
  Dataset folder is located at:
  `/models_and_data/taxonomy_and_origins/`
- **Dataset 2** - Trained word2vec models, gene embeddings, and classifier cross-validation evaluation (including ROC-AUC). Dataset folder is located at:
  `/models_and_data/cross_validation/`
  with classifier information at the sub-directory:
  `/LOTGOCV/label/paper_model/`
- **Dataset 3** - '.txt' corpus files.
  Dataset is located at:
  `/models_and_data/corpus.tar.gz`
- **Dataset 4** - Benchmark and optimization of performance and running time.
  Dataset folder is located at:
  `/models_and_data/benchmark and optimization/`
- **Dataset 5** - Gene families mapping to recently discovered anti-phage systems.
  Dataset folder is located at:
  `/models_and_data/novel_defense_mapping/`
- **Dataset 6** - Extened information about the novel systems found by our approach. This includes gene families '.faa' files and all domains found using HHpred. Dataset folder is located at:
  `/models_and_data/new_systems/`
- **Dataset 7** - Hypothetical words with a hit in NCBI NR protein database: The table contains the hypothetical words with a reliable prediction that were mapped to NR, but not to KEGG. Dataset is located at:
  `/models_and_data/hypothetical_in_NR_predictions_by_class.xlsx`
- **Dataset 8** - Word to label mapping.
  Dataset is located at:
  `/models_and_data/word_to_label.csv`

References

1. Mikolov, T., Sutskever, I., Chen, K., Corrado, G. S. & Dean, J. Distributed representations of words and phrases and their compositionality. *Advances in neural information processing systems* **26** (2013).
2. Mikolov, T., Chen, K., Corrado, G. & Dean, J. Efficient estimation of word representations in vector space. *arXiv preprint arXiv:1301.3781* (2013).
3. Levy, O. & Goldberg, Y. Linguistic regularities in sparse and explicit word representations. In *Proceedings of the eighteenth conference on computational natural language learning*, 171–180 (2014).
