# Supplementary material for: Deciphering microbial gene function using natural language processing
Source: Nat Commun. 2022 Sep 29;13:5731. doi: 10.1038/s41467-022-33397-4 (PMC9523054; doi:10.1038/s41467-022-33397-4)
Supplement: Supplementary file 2 — Reporting Summary [file 41467_2022_33397_MOESM2_ESM.pdf]

nature portfolio

Corresponding author(s): David Burstein

Last updated by author(s): Sep 10, 2022

Reporting Summary

Nature Portfolio wishes to improve the reproducibility of the work that we publish. This form provides structure for consistency and transparency in reporting. For further information on Nature Portfolio policies, see our Editorial Policies and the Editorial Policy Checklist.

Statistics

For all statistical analyses, confirm that the following items are present in the figure legend, table legend, main text, or Methods section.

n/a	Confirmed	
☐	☒	The exact sample size (*n*) for each experimental group/condition, given as a discrete number and unit of measurement
☒	☐	A statement on whether measurements were taken from distinct samples or whether the same sample was measured repeatedly
☒	☐	The statistical test(s) used AND whether they are one- or two-sided<br>*Only common tests should be described solely by name; describe more complex techniques in the Methods section.*
☒	☐	A description of all covariates tested
☒	☐	A description of any assumptions or corrections, such as tests of normality and adjustment for multiple comparisons
☐	☒	A full description of the statistical parameters including central tendency (e.g. means) or other basic estimates (e.g. regression coefficient) AND variation (e.g. standard deviation) or associated estimates of uncertainty (e.g. confidence intervals)
☒	☐	For null hypothesis testing, the test statistic (e.g. *F*, *t*, *r*) with confidence intervals, effect sizes, degrees of freedom and *P* value noted<br>*Give P values as exact values whenever suitable.*
☒	☐	For Bayesian analysis, information on the choice of priors and Markov chain Monte Carlo settings
☒	☐	For hierarchical and complex designs, identification of the appropriate level for tests and full reporting of outcomes
☒	☐	Estimates of effect sizes (e.g. Cohen's *d*, Pearson's *r*), indicating how they were calculated

Our web collection on statistics for biologists contains articles on many of the points above.

Software and code

Policy information about availability of computer code

Data collection	Gene prediction was performed using the Prokka pipeline (version 1.14.6) with prodigal version 3.0.0.<br>Removal of duplicate genomes was done by choosing genomes from Uniprot's non-redundant proteomes (https://www.uniprot.org/help/proteome_redundancy)<br>Metagenome redundant contigs were filtered using BBMAP dedupe utility (version 38.69).<br>All proteins were clustered first using CD-HIT version 4.6 and MMseq2 version bad16c765aac60d84a8fde3548adbb06b34980bd.<br>Alignment of clusters with more than 5 sequences by MAFFT version 7.475.<br>HMM profiles were created by hmmbuild from HMMer suite version 3.3.2.<br>proteins with no annotation in KEGG's database were searched against NCBI's non-redundant protein database (NR) downloaded on March 8, 2020 using DIAMOND version 2.0.1168.
Data analysis	All models used for training and scripts used for analysis are available in the repository https://github.com/burstein-lab/genomic-nlp, including links to the relevant data and code used for generating all paper figures.<br>All scripts are written in python 3.7, except the word2vec model which was applied using the gensim package in python version 3.8.3.<br>Deep neural networks were conducted in tensorflow version 2.2.0<br>Machine learning models for performance comparison were trained using sklearn package version 0.24.1 and xgboost version 1.3.3.<br>All figures were generated with seaborn package version 0.10.0 and matplotlib version 3.3.4.<br>Additional python package used were numpy version 1.20.1 and scipy version 1.2.1.<br>Full description of packages and versions are listed in the repository.<br><br>Benchmarking was performed using HHblits version 3.3.0 , HMMer version 3.3.2 and PSI-BLAST with BLAST version 2.7.1.

Searching for domains and remote homology for selecting candidate systems was performed using HHpred38 version 57c8707149031cc9f8edceba362c71a3762bd bf8 with default parameters against the databases: PDB mmCIF70 12Oct, Pfam-A v35, NCBI Conserved Domains(CD) v3.18, and TIGRFAMs v15.0.

For manuscripts utilizing custom algorithms or software that are central to the research but not yet described in published literature, software must be made available to editors and reviewers. We strongly encourage code deposition in a community repository (e.g. GitHub). See the Nature Portfolio guidelines for submitting code & software for further information.

Data

Policy information about availability of data

All manuscripts must include a data availability statement. This statement should provide the following information, where applicable:

- Accession codes, unique identifiers, or web links for publicly available datasets
- A description of any restrictions on data availability
- For clinical datasets or third party data, please ensure that the statement adheres to our policy

All data in this study was download directly from NCBI WGS and EBI Mgnify on March 14, 2020.
KEGG database was downloaded on on May 14, 2021.
The raw data generated in this study have been deposited in the Zenodo database under accession code 10.5281/zenodo.7047944 (https://zenodo.org/record/7047944). These data include representative sequences for each gene family, raw corpus text files, hypothetical family function prediction, gene family mappings to recently reported defense systems, and information on the putative new systems identified in this study.

Human research participants

Policy information about studies involving human research participants and Sex and Gender in Research.

Reporting on sex and gender	N/A
Population characteristics	N/A
Recruitment	N/A
Ethics oversight	N/A

Note that full information on the approval of the study protocol must also be provided in the manuscript.

Field-specific reporting

Please select the one below that is the best fit for your research. If you are not sure, read the appropriate sections before making your selection.

☒ Life sciences ☐ Behavioural & social sciences ☐ Ecological, evolutionary & environmental sciences

For a reference copy of the document with all sections, see nature.com/documents/nr-reporting-summary-flat.pdf

Life sciences study design

All studies must disclose on these points even when the disclosure is negative.

Sample size	Dataset included 596,338 non-redundant genomes and 22,923 metagenomes<br>These genomes and metagenomes include all publicly available in NCBI WGS on March 14, 2020 that passed the criteria described below, excluding genomes from the following taxonomic groups: Metazoa, Fungi, and Viridiplantae.<br>The final data size was 360,039,110 genes clustered into 563,589 families (unique “words”).
Data exclusions	Redundant genomes, that do not appear in Uniprots non-redudant proteome database (https://www.uniprot.org/help/proteome_redundancy) were fitered out.<br>Metagenome redundant contigs were filtered using BBMAP dedupe utility (version 38.69).<br>To allow for suffecient genomic context, contigs shorter than 10 kbp were filtered out, and so were genes with less than four<br><br>The number of genes was then reduced by the pre-processing for our model. Rare gene families (tokens) were filtered out with a threshold of at least 24 appearances per family. Ubiquitous tokens that appeared in a frequency greater than 10^-3 were also filtered out of the corpus, resulting a corpus size of 360,039,110 genes represented by 563,589 unique “words”.
Replication	Not applicable for this study, no experiments were performed.
Randomization	Bootstrapping and sub-sampling of genes as part of the rarefaction analysis was executed using a random generator from the numpy python package.<br>Also, for hyper-parameter tuning samples were randomized using K-fold cross validation from the sklearn package.

Blinding	Not applicable for this study, no experiment were performed.<br>Label assignment for genes was based on existing databases and well-defined ontologies (KEGG- Kyoto Encyclopedia of Genes and Genomes)

Reporting for specific materials, systems and methods

We require information from authors about some types of materials, experimental systems and methods used in many studies. Here, indicate whether each material, system or method listed is relevant to your study. If you are not sure if a list item applies to your research, read the appropriate section before selecting a response.

Materials & experimental systems

n/a	Involved in the study	
☒	☐	Antibodies
☒	☐	Eukaryotic cell lines
☒	☐	Palaeontology and archaeology
☒	☐	Animals and other organisms
☒	☐	Clinical data
☒	☐	Dual use research of concern

Methods

n/a	Involved in the study	
☒	☐	ChIP-seq
☒	☐	Flow cytometry
☒	☐	MRI-based neuroimaging
